# Buying to Cope With Scarcity During Public Emergencies: A Serial Mediation Model Based on Cognition-Affect Theory

**DOI:** 10.3389/fpsyg.2021.791850

**Published:** 2022-01-27

**Authors:** Xinran Ma, Jiangqun Liao

**Affiliations:** Department of Psychology, Tsinghua University, Beijing, China

**Keywords:** public emergency, panic buying, scarcity, perceived control, panic

## Abstract

Panic buying is a common phenomenon that occurs during public emergencies and has a significant undesirable impact on society. This research explored the effect of scarcity on panic buying and the role of perceived control and panic in this effect through big data, an online survey and behavior experiments in a real public emergency (i.e., COVID-19) and simulative public emergencies. The findings showed that scarcity aggravates panic buying (Studies 1–3), and this aggravation effect is serially mediated by perceived control and panic (Studies 2–3). Moreover, this serial mediation model is more suitable for public health emergencies (Study 3). These findings enrich the understanding of panic buying and provide important enlightenment for guiding rational public behavior and managing public opinion during public emergencies.

## Introduction

Panic buying refers to when consumers purchase an extraordinary number of items to cope with the probability of future shortages before or during a disaster or perceived disaster (Islam et al., 2020; Yuen et al., 2020; Herjanto et al., 2021). This phenomenon was globally witnessed in different countries or regions following the outbreak of COVID-19, as well as during many historical natural disasters and health crises such as SARS (Loxton et al., 2020; Li et al., 2021). This behavior has essential negative effects on social stability because it harms the balance of the supply chain, inflates prices and hinders vulnerable groups from obtaining protective resources (Yuen et al., 2020). However, at present, the empirical research on the causes and psychological mechanisms of this phenomenon during public emergencies is scarce and scattered (Garbe et al., 2020; Yuen et al., 2020; Li et al., 2021). Therefore, it is vital to further determine what happens behind panic buying, especially in the context of public emergencies.

To bridge this gap, the current paper started from a crucial variable that affects consumer behavior—scarcity (Hamilton et al., 2019), and developed a cognitive-affective serial model to explain the effect of scarcity on panic buying.

Scarcity implies a real or perceived state of having less than is needed (Shah et al., 2012; Hamilton et al., 2019). Specifically, in consumption, scarcity is defined as “a real or perceived threat to the consumer’s ability to meet their needs and desires due to a lack of, or a lack of access to, goods, services or resources” (Hamilton et al., 2019). Preventing and solving scarcity is an important purpose of panic buying during public emergencies (Yuen et al., 2020). From this perspective, scarcity may play a significant role in the cause of panic buying. Some studies, although only a few, have provided evidence for this suggestion. Limited time and limited quantity strengthen consumers’ urgency to buy (Gupta and Gentry, 2016) and hoarding (Gupta and Gentry, 2019). Anticipating food shortages improves consumers’ acceptance of food prices and incurs hoarding (Yangui and Aoud, 2015). The purchaser also increases hoarding to mitigate the negative impact of supply shortages when there is a shortage crisis in the supply chain (Yoon et al., 2018). These studies in daily consumption situations suggest that scarcity is one of the antecedents of panic buying.

Noteworthy, while hoarding consists of not only excessive acquisitions but also difficulty discarding (Cannito et al., 2021), the term “hoarding” in the mentioned studies mainly represents excessive acquisitions. Although excessive acquisition is a main behavioral characteristic of panic buying (Yuen et al., 2020; Herjanto et al., 2021; Li et al., 2021; Omar et al., 2021; Yoshino et al., 2021), excessive acquisitions in the daily context and panic buying during public emergencies are different in many aspects, such as behavior context, consumption objects, motivation and magnitude. Panic buying is a kind of herd behavior (Gao and Liu, 2017; Di Crosta et al., 2021) aiming at essential products and necessities to cope with crises (Ballantine et al., 2014; Herjanto et al., 2021; Yoshino et al., 2021) based on utilitarian motivation (Ballantine et al., 2014), whereas daily excessive acquisition is more individual according to people’s own interests based on both utilitarian and hedonic motivation (Islam et al., 2020). Particularly, a recent study clarified that, during the first peek of COVID-19 in Italy, the level of spending on necessities increased far more than that on non-necessities (Di Crosta et al., 2021), which suggested the consumer behavior changes more in necessities during emergencies and provided evidence for the notion that panic buying often occurs in necessities. Therefore, considering the different psychological antecedents of the utilitarian shopping (i.e., necessities products) and hedonic shopping (i.e., non-necessities products) (Di Crosta et al., 2021), further exploration of the effect of scarcity on panic buying during public emergencies and the mechanism is still needed.

To address this issue, we draw on the standard learning hierarchy model of consumption decision making (Lee and Goudeau, 2014) and the cognition-affect-coping model of coping behavior (Jung and Park, 2018). The standard learning hierarchy model illustrates a cognition-affect-behavior approach to consumers’ decision making (Oliver, 1999; Lee and Goudeau, 2014). For instance, beliefs about health and ecological welfare benefits have been shown to enhance affects in the form of hedonic attitudes and then improve attitudinal loyalty and behavioral loyalty to organic food (Lee and Goudeau, 2014). As a kind of consumption behavior, panic buying may also be serially impacted by cognition and affect.

Panic buying is also a kind of coping behavior exhibited during public emergencies (Bacon and Corr, 2020; Islam et al., 2020; Yuen et al., 2020). People generate a cognition-affect-coping model when facing threats and pressure; that is, an individual’s cognition and judgment of risk stimuli produce a corresponding affect and then influence the individual’s response behavior (Jung and Park, 2018). For example, when people perceive privacy threats, they feel angry and anxious and then refuse or restrict the use of private data by applications and complain about developers (Jung and Park, 2018). Hence, in view of coping, cognitive and affective factors still have vital effects on panic buying.

According to these models, the impact of scarcity on panic buying should also have a cognition-affect process. Specifically, perceived control as a cognitive factor and panic as an affective factor come to the surface.

Perceived control, which refers to the cognition that one can impact the environment and event results (Chen et al., 2017), is the most important, common and basic need for dealing with stress or traumatic events (Frazier et al., 2011; Kemp et al., 2014). Acquiring or strengthening one’s perceived control is the main motivation and goal of individuals when making decisions during public emergencies (Atalay and Meloy, 2020). According to compensatory control theory, people restore and rebuild their perceived control in other ways when their perceived control is threatened (Kay et al., 2010). During public emergencies, individuals produce compensatory consumption behaviors such as impulsive consumption to obtain perceived control (Sneath et al., 2009; Li et al., 2020). Panic buying also has a compensatory function, which allows individuals to regain control over their freedom (Gupta and Gentry, 2016) and defend against perceived risk (Li et al., 2021). Thus, perceived control appears to be a predictor of panic buying during public emergencies.

At the same time, scarcity has been shown to impair perceived control. For example, people experiencing the scarcity of material resources have a hard time resisting risks; thus, scarcity increases their perception of environmental uncertainty (Kraus et al., 2009). Moreover, the shortage of relevant information also reduces their perceived control over corresponding events (Yuen et al., 2020). Taken together, these studies indicate that perceived control mediates the effect of scarcity on panic buying.

Panic, which is characterized by anxiety and fear, is one of the main antecedents of panic buying (Tsao et al., 2019; Yuen et al., 2020). Panic directly drives self-protection behavior as a functional projection of individual self-protection motivation in a stress state (Maner et al., 2005). Hoarding the necessities of life in anticipation of or during a crisis is an adaptive survival strategy (Bentall et al., 2021) that addresses the possible shortage of resources (Gao and Liu, 2017) and reduces harm to health and property (Fung and Loke, 2010). Moreover, panic buying brings a temporary sense of security to individuals and relieves their sense of pressure (Sneath et al., 2009; Sterman and Dogan, 2015). Recent studies carried out during the early phase of the COVID-19 outbreak verified that anxiety is positively related to more overpurchasing (Garbe et al., 2020; Bentall et al., 2021).

At their root, anxiety and fear can be traced to scarcity (Islam et al., 2020; Omar et al., 2021). The scarcity felt during public emergencies emphasizes the lack of resources to help one resist threats and thus strengthens the perception of risk. These objective and subjective threats, which serve as information to hamper the possibility of survival and reproduction, stimulate individual self-protection motivation and ultimately result in panic (Maner et al., 2005). Therefore, panic explains the effect of scarcity on panic buying through the affective channel.

Moreover, perceived control and panic not only play solo roles but also have sequential roles. Low levels of perceived control encourage people to rethink and imagine various scenes, which then causes fear and anxiety about the unknown future (Kemp et al., 2014; Sterman and Dogan, 2015).

**FIGURE 1:**
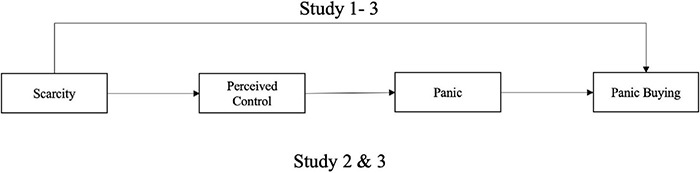
The overall framework.

In summary, during public emergencies, scarcity affects panic buying through a cognitive-affective path; that is, scarcity reduces the level of perceived control, which intensifies panic, and this increase in panic further strengthens panic buying (see Figure 1). To verify this model, this paper conducted three studies. The recent COVID-19 pandemic is a typical public emergency and thus provides a natural field experiment setting for this study. Hence, Study 1 and Study 2 were conducted during the early breakout of this real public emergency in China through the use of big data and an online survey, respectively, to explore the relationship between scarcity and panic buying. In addition, Study 3 was carried out to further test the effect of scarcity on panic buying causally in simulated different types of public emergencies and to explore the universality and boundaries of the theoretical model.

## Study 1

Study 1 aimed to preliminarily explore the relationship between scarcity and panic buying using big data from Chinese online users during the early period of the COVID-19 pandemic. During the home quarantine phase in the early outbreak period, the usage time and number of active users of China mobile internet increased significantly,^1^ which suggests that Chinese emotional and behavior trajectories on the internet increased significantly. Thus, it is feasible to observe Chinese attitudes and behaviors during the COVID-19 outbreak through the use of these online big data.

Previous studies have taken cultural products (such as words and names related to cultural values in books) as “out of mind” indicators (i.e., objective indicators) to reflect the values or cultural tendencies of groups or times (Grossmann and Varnum, 2015). According to this approach, network information containing keywords related to scarcity and panic buying, such as news, new media soft articles, and users’ publishing content in social networks, is also an objective embodiment of public attitudes and behaviors in social life. The amount of information represents the intensity of corresponding attitudes and behaviors. Therefore, Study 1 used the number of information containing keywords related to scarcity and panic buying as the indicators of scarcity and panic buying, respectively.

### Methods

The amount of information that contained corresponding keywords related to scarcity and panic buying every day during the most serious stage of the COVID-19 pandemic in China (i.e., January 21, 2020 to April 9, 2020) was obtained from 11 channels (including web pages, WeChat, Weibo, APP, online forum, newspapers, short video platforms, TouTiao, Sohu, online question-and-answer website and other online platforms)^2^ through a Chinese data platform.^3^ Two steps were involved in this process.

#### Step 1: The Selection of Keywords

##### Scarcity

Based on the definition of scarcity, that is, “having less than is needed” (Shah et al., 2012), five groups of words describing shortages in the COVID-19 pandemic in Chinese, such as “yi qing (referring to ‘COVID-19’ in Chinese) and duan que (referring to ‘shortage’ in Chinese),” were taken as keywords for scarcity. We set “or” as the parallel logic between each group of keywords and “and” as the logic between the two words of each group of keywords. That is, the information was required to contain at least one group of keywords and the two words involved in one group of keywords at the same time.

##### Panic Buying

Since “qiang gou” is a more common word used by Chinese media and people to express panic buying, it was used as the keyword for panic buying. The key materials for epidemic prevention and control,^4^ such as medicinal alcohol, masks, and medicine, were also included in the word groups to ensure the relevance of the information to the epidemic. Finally, ten groups of keywords about panic buying were applied; the example items were “qiang gou (referring to panic buying in Chinese) and kou zhao (referring to masks in Chinese).” Likewise, the two words in the same group were based on “and” logic, whereas each group of keywords followed “or” logic.

#### Step 2: Data Preprocessing

Data preprocessing, including the following two steps, was carried out to denoise the obtained information. First, the platform’s de-duplication function was used to exclude information from different media channels that had duplicate or similar content. Second, since scarcity and panic buying are negative events, only the information that reflected negative emotions was retained according to the emotional attributes of each piece of information provided by the platform. After preprocessing, a total of 218,953 pieces (2,270,178 pieces before preprocessing) of scarcity-related information and 116,593 pieces (1,761,705 pieces before preprocessing) of panic buying-related information were obtained.

### Results

**FIGURE 2:**
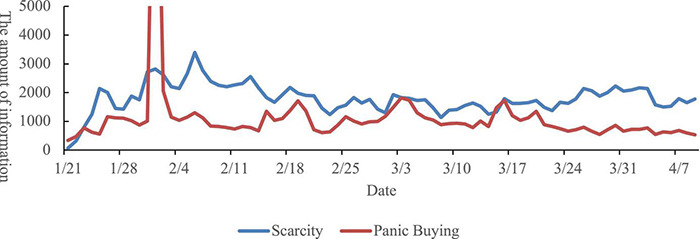
The trend of the amount of information.

Figure 2 shows the covariant trend of the amount of scarcity-related information and the amount of panic buying-related information. A linear regression analysis performed by SPSS 26.0 showed that the amount of information about scarcity was marginally positively related to the amount of information about panic buying, β = 0.21, *t*(78) = 1.92, *p* = 0.059, 95%CI (−0.02, 0.94). And the significant positive relation existed in every channel independently (βs = 0.30–0.48, *ts* = 2.82–4.89, *ps* ≤ 0.001) except in the online question-and-answer website [β = −0.05, *t*(78) = −0.40, *p* = 0.693] (see Table 1).

**TABLE 1:** Summary of regression analysis of panic buying to scarcity for different channels (*N* = 80).

	All channels	1	2	3	4	5	6	7	8	9	10	11
Scarcity	0.21^†^	0.39***	0.48***	0.43***	0.41***	0.39***	0.44***	0.30**	0.38***	0.36**	−0.05	0.40***
*F*	3.67^†^	13.59***	23.86***	17.71***	15.92***	13.86***	18.66***	7.94**	13.52***	11.51**	0.16	15.24***
*R*	0.21	0.39	0.48	0.43	0.41	0.39	0.44	0.30	0.41	0.36	0.05	0.40
Source proportion-Scarcity		13.32%	27.03%	19.50%	13.20%	1.80%	0.49%	0.32%	8.53%	4.82%	1.62%	9.39%
Source proportion-Panic buying		30.94%	25.14%	13.46%	8.94%	1.71%	0.23%	0.74%	5.83%	3.26%	3.12%	6.61%

*1 = Weibo, 2 = WeiChat, 3 = APP, 4 = web pages, 5 = online forum, 6 = newspapers, 7 = short video platforms, 8 = TouTiao, 9 = Sohu, 10 = online question-and-answer website, and 11 = other online platforms.*

***p < 0.001, **p < 0.01, *p < 0.05, ^†^p < 0.10, the same below.

*Source proportion refers to the proportion of information quantity of each channel in the total information quantity.*

### Discussion

Study 1 initially illustrated the positive correlation between scarcity and panic buying during a real public emergency using the amount of information as the objective indicator of scarcity and panic buying at the group level. To further test the relationship between scarcity and panic buying and, more importantly, to investigate the psychological mechanism behind the relationship, Study 2 was conducted using individual self-report indicators. In addition, considering that news mostly reflects objective phenomena in social life, Study 1 paid attention to objective scarcity. However, scarcity is a multifaceted concept that contains both objective and subjective aspects (Hamilton et al., 2019). Therefore, referring to previous research (Piff et al., 2010), we decided to develop Study 2 from subjective scarcity.

## Study 2

Study 2 was designed to examine the effect of scarcity on panic buying again, along with the serial mediating role of perceived control and panic, through a nationwide online survey administered during the early period of the COVID-19 outbreak in China.

### Methods

#### Participants and Procedure

According to Fritz and Mackinnon (2007), a sample size of at least 462 is necessary to detect a small effect in both the pathway between the independent variable and the mediator and the pathway between the mediator and the dependent variable, under 0.8 power, using the bias-corrected bootstrap test to estimate the indirect effect. A total of 658 samples (234 males, *M*_age_ = 32.94, *SD* = 11.92) from 29 provinces in mainland China and Hong Kong, Macao and Taiwan regions were collected through an online survey conducted from the 11th to the 20th of February 2020. Approximately 83.7% of the respondents reported having a college degree or above. Moreover, 94.4% of the participants reported being healthy, and 88.8% of participants reported that there were no cases of infection in the community where they lived.

The survey was distributed on various platforms, including WeChat, Weibo, QQ, etc. After the participants clicked on the survey hyperlink, they could read the electronic informed consent following the Declaration of Helsinki’s ethical standards and approved by Institution Review Board for Human Participants at the university where authors are, which clearly explained the aims and the procedure of the study and the participants’ rights and reward. Participants then chose “agree” or “disagree” to participate, according to their own wishes. Each participant who completed the survey eventually received 3 Chinese yuan as a reward.

#### Measures

##### Perceived Scarcity

Perceived scarcity was evaluated with four self-developed items. The four items are “The prevention measures against COVID-19 I adopt,” “The prevention equipment against COVID-19 I have,” “The knowledge about epidemic prevention I know,” and “The useful information about COVID-19 I know.” The participants used a 7-point Likert scale (ranging from 1 = very sufficient to 7 = very insufficient) to rate each item. A higher mean score indicated a stronger scarcity perception. The Cronbach’s α of these items was 0.74 in the present study, and the exploratory factor analysis (CFA) showed a one-factor structure that explained 57.57% of the total variance (see Supplementary Material for more details of CFA at: https://osf.io/sfj52/?view_only==9574c3d59a5e4d8c94dac620afdb416b).

##### Panic Buying

Panic buying was measured by a behavioral indicator, namely, payment degree, which depicted the highest price that people were willing to pay for a good or resource. Although hoarding or over-purchasing is the key behavioral characteristic of panic buying, in view of the performance of panic buying in real life, panic buying is reflected not only in the quantity of consumption but also in the acceptance of prices or willingness to pay (Yangui and Aoud, 2015). In addition, since some epidemic prevention materials were still in shortage at the time of our survey, people could not hoard a large number of them but could obtain them at any cost. Therefore, we detected panic buying *via* payment degree in study 2.

In this part, the participants were shown four popular pieces of epidemic prevention equipment, including masks, alcohol, disinfectant and cold medicine, and then decided how much they were most willing to pay for these items at the moment. The more money that the participants were willing to pay, the greater the cost they were willing to pay for resources and the greater their urgency of obtaining resources. The payment amount of the four materials was standardized and averaged to develop the overall payment degree.

##### Perceived Control

The 8-item perceived present control subscale of the Perceived Control Over Stressful Events Scale (Frazier et al., 2011) was used to assess perceived control. Perceived present control estimates the individual’s perceived control over the current events and reflects the individual’s general beliefs that they can better control important results (Frazier et al., 2011). An example item is “How I deal with this event is now under my control.” The participants were asked to rate each item on a scale ranging from 1 (very strongly disagree) to 4 (very strongly agree) based on their feelings about COVID-19. A higher mean score indicated a stronger level of perceived control. The Cronbach’s α of these items was 0.73 in the present study.

##### Panic

Four items from the negative affect subscale of the Positive and Negative Affect Schedule (Watson et al., 1988) were used to assess panic, that is, “scared,” “afraid,” “nervous,” and “jittery.” The items were rated on a scale ranging from 1 (very slightly or not at all) to 5 (extremely) based on the extent to which the participants had felt those emotions over the past 2 weeks. A higher mean score indicated a stronger panic. The Cronbach’s α of these items was 0.88 in the present study.

##### Demographic

Several demographic variables were measured as covariates, including biological gender (1 = males, 0 = females), age, per capital monthly household income and physical (1 = there is a confirmed or suspected case at home, 7 = there is a confirmed or suspected case in the province) and psychological distance (1 = very close, 7 = very distant) from COVID-19.

### Results

#### Common Method Bias

The data for Study 2 were collected completely by the self-reported method. Scholars have proposed that when data are collected from a single source, there is the possibility of common method bias (CMB) in the dataset (Podsakoff et al., 2012). To reduce concerns over CMB, as suggested by Podsakoff et al. (2003), we conducted Harman’s single-factor test to check if CMB was an issue in the current study. The results of the Herman test revealed that a single factor has a value of variance of less than 50% (unrotated: 29.42%, rotated: 18.62%). In addition, Table 1 indicates that the intercorrelation of all the constructs was less than 0.90 (Pavlou and El Sawy, 2006). These findings indicated that CMB was not a major issue in the present study.

#### Hypothesis Testing

A correlation analysis conducted on the variables of interest showed significant correlations between all measured constructs (see Table 2). Hayes’ PROCESS macro (version 3.4, Model 6, bootstrapping *N* = 5000) was then employed to conduct a regression-based serial mediation model to further investigate the association between perceived scarcity and panic buying, as serially mediated by perceived control and panic, taking gender, age, income (after logarithm transformation to correct the skew distribution) and distance from COVID-19 as covariates.

**TABLE 2:** Descriptive statistics and correlations among variables assessed in Study 2.

	*M* ± *SD*	Perceived scarcity	Panic buying	Perceived control	Panic
Perceived scarcity	2.91 ± 0.84	0.74			
Panic buying	0.03 ± 0.89	0.08*	NA		
Perceived control	2.81 ± 0.50	−0.30***	−0.08*	0.73	
Panic	2.77 ± 0.98	0.19**	0.11**	−0.52***	0.88

*The numbers on the diagonal are Cronbach’s α.*

***p < 0.001, **p < 0.001, *p < 0.05.

**FIGURE 3:**
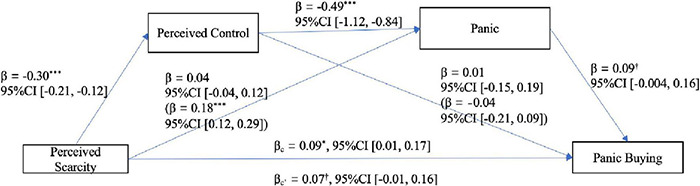
The serial mediation model of Study 2. The parameters in parentheses are total effects. ***p < 0.001, *p < 0.05, ^†^*p* < 0.10.

After controlling for all covariates, under the significance level of 0.05, a significant serial mediating effect of perceived control and panic (i.e., perceived scarcity → panic → perceived control → panic buying) was found (0 was not included in the 95% confidence interval, 95%CI), and the direct effect of perceived scarcity became smaller and marginally significant when the indirect effects were separated from the total effect. However, the independent mediating effects of perceived control and panic were not proven in the whole serial mediation model. When taken as mediation separately, panic played a mediating role, whereas perceived control did not (see Table 3 and Figure 3). In addition, the possible competitive model (i.e., perceived scarcity → panic → perceived control → panic buying) was not supported [*Effect* = −0.001, *SE* = 0.004, 95%CI (−0.01, 0.01)].

**TABLE 3:** Summary of indirect effects in study 2.

Indirect effects	Effect (SE)	95%CI
Total (for serial mediation model)	0.014 (0.01)	[−0.01,0.04]
perceived scarcity → perceived control → panic buying	−0.003(0.02)	[−0.03,0.03]
perceived scarcity → panic → panic buying	0.003 (0.004)	[−0.004,0.01]
perceived scarcity → panic → perceived control → panic buying	0.013 (0.01)	[0.001,0.03]
perceived scarcity → perceived control → panic buying (singlemediation model)	0.01 (0.01)	[−0.01,0.04]
perceived scarcity → panic → panic buying (single mediation model)	0.02 (0.01)	[0.002,0.03]
perceived scarcity → perceived control → panic (single mediation model)	0.15 (0.02)	[0.11,0.19]
perceived control → panic → panic buying (single mediation model)	−0.05(0.02)	[−0.09,−0.01]
		

### Discussion

Study 2 again demonstrated the aggravating effect of scarcity on panic buying from the perspective of perceived scarcity and verified that this effect is serially mediated by perceived control and panic.

It is worth noting that perceived control has never played an independent mediating role, whether as a serial mediator or as a mediator alone, because it has no effect on panic buying. However, herein, perceived control indirectly affected panic buying through panic [95%CI (−0.09, −0.004)]. These results suggest that the mechanism by which perceived control affects panic buying could be different from that by which it affects other consumption behaviors such as impulsive consumption (Sneath et al., 2009; Li et al., 2020). While this mechanism may not be based on the need to compensate control, it may exist because low perceived control intensifies individual panic and panic then exacerbates panic buying. On the other hand, panic played a complete mediating role when it was used as a mediator alone. However, when perceived control was included as a mediator at the same time, this mediation disappeared because the effect of scarcity on panic was completely mediated by perceived control [95%CI (0.11, 0.19)] at this time, which led to the insignificant direct effect of scarcity on panic. This result implies that the effect of scarcity on panic is realized through perceived control. To summarize the above results, in the “black box” of scarcity affecting panic buying, the serial mediation of perceived control and panic is more essential and stable.

Study 1 and Study 2 both tested the effect of scarcity on panic buying using big data and an online survey, respectively, but they did not check the causal link between these two variables (Kofta et al., 2020). Meanwhile, since the first two studies are conducted in the same context of COVID-19—a real public emergency—more studies are needed to examine the existing findings during other public emergencies to explore the generalization and boundaries of the current model (Zhou et al., 2019; Kofta et al., 2020). Therefore, Study 3 was performed with the paradigm of priming to examine the causal link between scarcity and panic buying in different public emergencies.

## Study 3

Study 3 concentrated on the causal link between scarcity and panic buying through three experiments regarding different public emergencies and the generalization and boundaries of the current results.

Public emergencies comprise four categories: natural disasters, accident calamities, public health emergencies, and social security emergencies (The State Council, 2006). In reality, panic buying usually occurs during hurricanes and public health emergencies (such as SARS, COVID-19 and nuclear leakage crises) (Loxton et al., 2020). Therefore, the experimental contexts of Study 3 were set around these public emergencies.

Study 3a was implemented to directly verify the existing results by priming scarcity in a simulated respiratory epidemic context, which was similar to COVID-19. Study 3b was designed to explore whether the existing results are specific to the epidemic situation in another public health emergency (i.e., pollutant leakage) that suggests serious harm to life and health and a long duration. Study 3c was executed to further examine whether the existing model was applicable to public emergencies other than public health emergencies, such as hurricanes, with a shorter duration than an epidemic and characteristics of forewarning (Kemp et al., 2014).

### Study 3a

#### Methods

##### Participants and Design

This study adopted a between-subjects single-factor design (primed scarcity: scarcity or non-scarcity). Using G*Power 3.1 (Faul et al., 2007), we determined that we required at least 172 samples under a sufficient power (1−β = 0.90) and the significance level of 0.05 to detect a medium-sized effect (*Cohen’s d* = 0.5), using *t*-tests to test the difference between two independent groups. Finally, a total of 252 Chinese adults (96 males, *M*_age_ = 21.91, *SD* = 2.59) recruited online through Sojump (a Chinese online data collection platform^5^) participated in this study, during the last week of June 2020 to the middle of August 2020. Every participant read the online informed consent similar to the one used in Study 2 and then decided whether to participant the study or not. The protocol was following the Declaration of Helsinki’s ethical standards and approved by Institution Review Board for Human Participants at the university where authors are. Everyone who completed the experiment received 3 Chinese yuan as a reward.

##### Procedure and Materials

The participants read two short paragraphs. The first paragraph outlined an assumed respiratory epidemic background, including the route of transmission, incidence pattern, risk of epidemic and possible prevention measures. The second paragraph referred to the experimental manipulation. For the scarcity group, the paragraph described the surge in demand for protective supplies and information related to the emergency and the shortage of inventory. For the non-scarcity group, the paragraph described the stable supply and demand of materials and information and the sufficient inventory (see more details in Supplementary Material at: https://osf.io/sfj52/?view_only==9574c3d59a5e4d8c94dac620afdb416b). The participants were randomly divided into the two experimental groups. After reading the two paragraphs, the participants were asked to accomplish the manipulation checks and questions about perceived control, panic, and panic buying in turn. To ensure that the participants carefully read all two paragraphs, the presentation length of time of this page was set to 60 s (Kofta et al., 2020).

##### Measures

*Manipulation Check.* Two items adapted from Kristofferson et al. (2017) were used as manipulation checks, namely, “How would you describe the quantity of the protective supplies in the above situation?” and “How would you describe the quantity of the information related to epidemic in the above situation?” Each item was rated on a scale ranging from 1 (very sufficient) to 7 (very insufficient). The individual items were averaged to create a composite scarcity score. Higher scores signaled a higher scarcity perception, whereas lower scores signaled a higher abundant perception.

*Panic Buying.* Two behavioral indicators were used to measure panic buying: payment degree and hoarding. For payment degree, the participants declared the highest price they were willing to pay for the four protective supplies previously mentioned in the reading material, namely, surgical masks, medical alcohol, cold medicine and basic food, after being reminded of the regular price of each item. The payment degree for each item was calculated by the subtraction of the payment price over the regular price [i.e., (payment price − regular price)/regular price]. Since the term basic food is a general name used for a class of commodities, and the regular price could not be set directly, the participants declared the highest percentage they were willing to pay more than usual for basic food, and this percentage was divided by 100 to obtain the payment degree of basic food. The payment degree for each commodity was then averaged to develop the overall payment degree.

For hoarding, the participants answered how many/much surgical masks/medical alcohol/cold medicine/basic food they planned to buy in the described situation. The overall hoarding index was obtained by standardizing and averaging the amount of hoarding for the four commodities.

*Perceived Control.* One item assessing perceived control in general was used to measure perceived control. The item was “How I deal with this epidemic is now under my control” (Frazier et al., 2011). The participants were asked to rate this item on a scale ranging from 1 (very strongly disagree) to 4 (very strongly agree). A higher mean score indicated a stronger perceived control.

*Panic.* The assessment of panic was identical to that used in Study 2. The Cronbach’s α of these items was 0.87 in the present study.

*Demographic.* Gender (1 = males, 0 = females), age, per capita monthly household income, physical distance from COVID-19 (1 = there is a confirmed or suspected case at home, 7 = there is a confirmed or suspected case in the province), physical health condition in COVID-19 (from 1 = uninfected to 4 = infected) and epidemic level of the place where participants were during the COVID-19 outbreak (1 = mildly affected area, 4 = central affected area) were measured as covariates.

#### Results

##### Preliminary Analysis

The participants perceived more scarceness in the scarcity condition than in the non-scarcity condition, which suggested the scarcity manipulation was successful. Furthermore, the results of *t*-tests showed the significant effects of primed scarcity on participants’ perceived control, panic and panic buying (see Table 4).

**TABLE 4:** Descriptive statistics of key variables and the effect sizes of scarcity manipulation in study 3.

Study	Variable	Scarcity manipulation	Payment degree	Hoarding	Perceived control	Panic
**Study 3a**	Scarcity group	6.04 ± 0.84	1.09 ± 0.70	0.11 ± 0.63	2.38 ± 0.73	3.45 ± 0.81
	Non-scarcity group	2.81 ± 1.00	0.67 ± 0.52	−0.11 ± 0.68	2.76 ± 0.64	2.93 ± 0.79
	*t* (250)	27.81	5.50	2.64	−4.43	5.19
	*p*	<0.001	<0.001	0.009	<0.001	<0.001
	*Cohen’s d*	3.52	0.70	0.33	0.56	0.66
**Study 3b**	Scarcity group	6.07 ± 0.76	1.30 ± 1.60	0.13 ± 0.76	2.08 ± 0.65	3.63 ± 0.74
	Non-scarcity group	2.51 ± 0.90	0.61 ± 0.98	−0.12 ± 0.67	2.67 ± 0.67	3.00 ± 0.84
	*t* (260)	34.57	4.23	2.80	−7.27	6.48
	*p*	<0.001	<0.001	0.005	<0.001	<0.001
	*Cohen’s d*	4.29	0.52	0.35	0.90	0.80
**Study 3c**	Scarcity group	5.62 ± 0.81	1.08 ± 1.36	0.24 ± 0.96	2.39 ± 0.64	3.11 ± 0.83
	Non-scarcity group	2.29 ± 0.71	0.58 ± 0.67	−0.24 ± 0.47	2.81 ± 0.37	2.74 ± 0.80
	*t* (254)	34.91	3.75	5.06	−4.89	3.65
	*p*	<0.001	<0.001	0.009	<0.001	<0.001
	*Cohen’s d*	4.38	0.47	0.64	0.61	0.46

*This table showed descriptive statistics of key variables and the effect sizes of scarcity manipulation in Study 3a, 3b, and 3c.*

##### Serial Mediation Analysis

**FIGURE 4:**
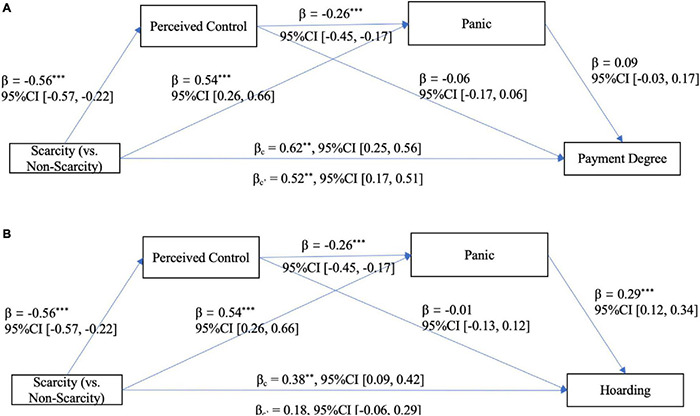
**(A, B)** The serial mediation model of Study 3a. ***p < 0.001, **p < 0.01.

Similar to Study 2, Hayes’ PROCESS macro (version 3.4, Model 6, bootstrapping *N* = 5000) was employed to conduct a regression-based serial mediation model. The results showed that, after controlling for all covariates, under the significance level of 0.05, for payment degree (see Figure 4A), a significant total effect and a significant direct effect of primed scarcity (0 = not scarcity group, 1 = scarcity group) were found. However, no significant indirect effect was found (see Table 5 and Figure 4A). In addition, the possible competitive model (i.e., perceived scarcity-panic-perceived control panic buying) was not supported [*Effect* = 0.01, *SE* = 0.01, 95%CI (−0.01, 0.04)].

**TABLE 5:** Summary of indirect effects in study 3a.

Indirect effects	Effect (SE)	95%CI
**Payment degree**		
Total	0.08 (0.06)	[−0.02, 0.20]
scarcity → perceived control → panic buying	0.03 (0.03)	[−0.015, 0.12]
scarcity → panic → panic buying	0.04 (0.03)	[−0.01, 0.11]
scarcity → panic → perceived control → panic buying	0.01 (0.01)	[−0.003, 0.04]
**Hoarding**		
Total	0.20 (0.07)	[0.08, 0.35]
scarcity → perceived control → panic buying	0.004 (0.04)	[−0.07, 0.10]
scarcity → panic → panic buying	0.16 (0.05)	[0.07, 0.27]
scarcity → panic → perceived control → panic buying	0.04 (0.02)	[0.01, 0.09]

For hoarding (see Figure 4B), the total effect of primed scarcity was significant, and it was serially mediated by perceived control and panic and independently mediated by panic, which made the direct effect of primed scarcity non-significant. In addition, the possible competitive model was not supported [*Effect* = 0.002, *SE* = 0.01, 95%CI (−0.02, 0.03)].

#### Discussion

Study 3a directly verified the causal link between scarcity and panic buying in a similar context as that of COVID-19. The serial mediating role of perceived control and panic was confirmed in the relationship between scarcity and hoarding. However, although participants primed with scarcity were willing to pay higher prices for protective supplies than their counterparts in the non-scarcity group, it seemed that this was not because scarcity reduced their perceived control and then intensified their panic. The possible reason is that the government’s price-limiting measures have strengthened people’s perceived control and reduced the panic about price increase.

Based on the results, to further certify the stability of the serial mediation model and explore whether this model is specific to the epidemic context, we carried out Study 3b in relation to another public health emergency (i.e., leakage crisis).

### Study 3b

#### Methods

##### Participants and Design

Study 3b adopted the same design as that used in Study 3a. A total of 262 Chinese adults (95 males, *M*_age_ = 22.35, *SD* = 2.90) recruited online during the first week of July 2020 to the middle of August 2020 through Sojump participated in this study. The participants read the online informed consent same as study 3a and obtained 3 Chinese yuan as a reward.

##### Procedure and Materials

Similar to Study 3a, the participants first read a paragraph depicting the leakage of radioactive pollutants, including the route, the degree, the risk and the protective measures of pollution. Then, they received a similar scarcity manipulation and answered questions similar to those used in Study 3a.

##### Measures

The assessments of the manipulation checks, perceived control, panic (Cronbach’s α = 0.87) and demographic variables^6^ were identical to those used in Study 3a. The indicators of panic buying remained payment degree and hoarding. However, the specific measurements had a few differences. To exclude baseline differences in individuals’ own reserve habits, hoarding was measured by the highest amount of emergency supplies (including bottled water, fruits and vegetables, basic food, antidotes and tickets) that the participants would stockpile more than usual in the given condition. The overall hoarding index was obtained by standardizing and averaging these amounts. Meanwhile, to rule out the influence of price anchoring and the participants’ own consumption levels, payment degree was measured by the highest percentage that the participants were willing to pay above the usual. These percentages were then divided by 100 and averaged to develop the overall payment degree.

#### Results

##### Preliminary Analysis

Participants in the scarcity group perceived higher scarceness, less control, more panic, and showed more panic buying than participants in the non-scarcity group (see Table 4).

##### Serial Mediation Analysis

**FIGURE 5:**
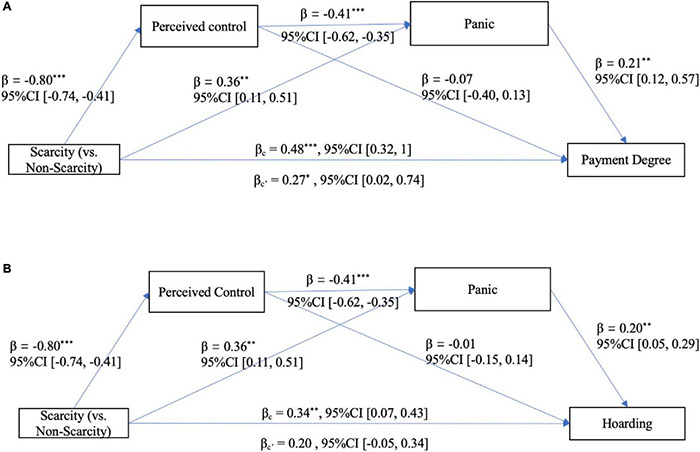
**(A, B)** The serial mediation model of Study 3b. ***p < 0.001, **p < 0.01, *p < 0.05.

Hayes’ PROCESS macro (version 3.4, Model 6, bootstrapping *N* = 5000) was employed to conduct the mediation analysis. After controlling for all covariates, under the significance level of 0.05, for payment degree (see Figure 5A), a significant total effect and a significant direct effect of primed scarcity (0 = not scarcity group, 1 = scarcity group) were found. And the serial mediating effect of perceived control and panic and the independent mediating effect of panic were also significant, whereas the independent mediating effect of perceived control was non-significant (see Table 6 and Figure 5A). In addition, the possible competitive model was not supported [*Effect* = 0.02, *SE* = 0.02, 95%CI (−0.02, 0.06)].

**TABLE 6:** Summary of indirect effects in study 3b.

Indirect effects	Effect (SE)	95%CI
**Payment degree**		
Total	0.20 (0.07)	[0.08, 0.34]
scarcity → perceived control → panic buying	0.06 (0.06)	[−0.05, 0.18]
scarcity → panic → panic buying	0.08 (0.03)	[0.02, 0.15]
scarcity → panic → perceived control → panic buying	0.07 (0.02)	[0.03, 0.13]
**Hoarding**		
Total	0.14 (0.06)	[0.02, 0.27]
scarcity → perceived control → panic buying	0.01 (0.06)	[−0.10, 0.11]
scarcity → panic → panic buying	0.07 (0.03)	[0.02, 0.14]
scarcity → panic → perceived control → panic buying	0.06 (0.02)	[0.02, 0.12]

For hoarding (see Figure 5B), similarly, a significant total effect of perceived scarcity, a significant serial mediating effect of control and panic and the independent mediating effect of panic were found, whereas the direct effect of primed scarcity was not significant when the indirect effects were separated from the total effect (see Table 6 and Figure 5B). In addition, the possible competitive model was not supported [*Effect* = 0.002, *SE* = 0.02, 95%CI (−0.04, 0.04)].

#### Discussion

Using a new public health emergency different from the epidemic scenario, Study 3b once again confirmed that scarcity exacerbates panic buying and that this aggravation is transmitted *via* reduced perceived control and intensified panic. These results indicate that the serial mediating pathway is not specific to epidemic-related emergencies but also applies to other public health emergencies.

Considering that people’s panic buying in real life is also common in some natural disasters, such as hurricanes, which are different from public health emergencies, with shorter durations and characteristics of forewarning and being of less threat to people’s lives and health (Kemp et al., 2014), Study 3c was implemented to further explore the generalization and boundary of the existing results in a simulated hurricane context.

### Study 3c

#### Methods

##### Participants and Design

Study 3c adopted the same design as that used in both Study 3a and Study 3b. A total of 256 Chinese adults (103 males, *M*_age_ = 21.40, *SD* = 2.25) recruited online during the middle of July 2020 to the middle of August 2020 through Sojump participated in this study. Similarly, the participants read the online informed consent form same as study 3a and obtained 3 Chinese yuan as a reward.

##### Procedure and Materials

The procedure and manipulation materials were similar to those used in both Study 3a and Study 3b.

##### Measures

The assessments of all the variables were identical to those used in Study 3b.^7^ The participants made consumption decisions regarding five emergency supplies, namely, bottled drinking water, fruits and vegetables, basic food, flashlights, and tickets, and the five commodities were presented at random.

#### Results

##### Preliminary Analysis

Participants in the scarcity group perceived higher scarceness, less control, more panic, and showed more panic buying than participants in the non-scarcity group (see Table 4).

##### Serial Mediation Analysis

**FIGURE 6:**
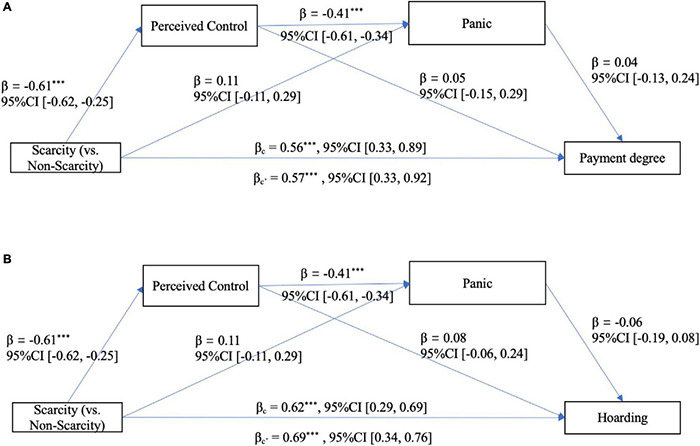
**(A, B)** The serial mediation model of Study 3c. ***p < 0.001.

Hayes’ PROCESS macro (version 3.4, Model 6, bootstrapping *N* = 5000) was employed to conduct the mediation analysis. After controlling for all covariates, for payment degree (see Figure 6A), a significant total effect and a significant direct effect of primed scarcity (0 = not scarcity group, 1 = scarcity group) was found, whereas no significant indirect effect was found (see Table 7 and Figure 6A). In addition, the possible competitive model was not supported [*Effect* = −0.01, *SE* = 0.01, 95%CI (−0.03 0.01)].

**TABLE 7:** Summary of indirect effects in study 3c.

Indirect effects	Effect (SE)	95%CI
**Payment degree**		
Total	−0.01 (0.04)	[−0.09, 0.06]
scarcity → perceived control → panic buying	−0.03 (0.04)	[−0.11, 0.04]
scarcity → panic → panic buying	0.004 (0.01)	[−0.01, 0.03]
scarcity → panic → perceived control → panic buying	0.01 (0.01)	[−0.02, 0.04]
**Hoarding**		
Total	−0.07 (0.04)	[−0.16, 0.02]
scarcity → perceived control → panic buying	−0.05 (0.05)	[−0.14, 0.04]
scarcity → panic → panic buying	−0.01 (0.01)	[−0.04, 0.02]
scarcity → panic → perceived control → panic buying	−0.01 (0.02)	[−0.06, 0.02]

Similar results were obtained for hoarding (see Table 7 and Figure 6B). In addition, the possible competitive model was not supported [*Effect* = −0.01, *SE* = 0.01, 95%CI (−0.04, 0.01)].

#### Discussion

Study 3c attempted to expand the existing findings of former studies to another type of public emergency that had a different nature from that of a public health emergency. The aggravating effect of scarcity on panic buying was repeated. Nevertheless, the “perceived control → panic” pathway was not tenable to explain this effect this time.

People’s risk assessment of crises usually consists of two aspects: dread and unknown (Slovic, 1987). Compared with public health emergencies, hurricanes/typhoons are more frequent and more common (low uncertainty). Thus, the related forewarning mechanisms and emergency plans are relatively mature. As a result, the threat to life is relatively controllable (low worry) in hurricanes/typhoons. Therefore, individuals’ risk assessments of hurricanes/typhoons and panic in this kind of crisis are lower than those found during public health emergencies.^8^ Therefore, low levels of panic have no impact on panic buying, which leads to the final failure of serial mediation in Study 3c.

In summary, Study 3 certified the causal link between scarcity and panic buying in different public emergencies and the psychological mechanism of this link and discovered the boundary of this mechanism. Specifically, the serial mediating pathway (i.e., perceived control → panic) only explains the aggravating effect of scarcity on panic buying during public health emergencies but not during public emergencies with lower risk levels, such as hurricanes.

## General Discussion

Panic buying, which is socially undesirable, is usually observed during public emergencies, such as natural disasters like hurricanes, and health crises like SARS and the recent COVID-19 outbreak (Loxton et al., 2020). Discovering the causes and underlying processes of this phenomenon is of paramount significance for both individuals and society. However, the scientific research on this topic is still in its infancy (Yuen et al., 2020; Li et al., 2021; Sherman et al., 2021) despite the increasing focus on panic buying during the recent COVID-19 outbreak. To address this issue, the current study focused on scarcity and explored how scarcity impacts panic buying from a cognition-affect pathway using big data, an online survey and behavioral experiments. Several valuable results were found.

First, the findings suggest that scarcity aggravates panic buying. Scarcity is vital to consuming behavior (Hamilton et al., 2019), and it always occurs during public emergencies. Some studies have previously discussed the effect of scarcity on hoarding (excessive acquisition) in the retail industry (Yangui and Aoud, 2015; Gupta and Gentry, 2016, 2019) and supply chains (Yoon et al., 2018). Although excessive acquisition is a key indicator of panic buying, excessive acquisition in the daily context and panic buying during public emergencies are different in many aspects, such as background, targets, motivation, and magnitude, which may lead to distinctive mechanisms. On the other hand, since the repeatability crisis of psychological research attracts attention, the background of the results and the extent to which the results can be generalized have become important concerns of researchers (Simons et al., 2017; Anderson et al., 2019). Therefore, while it is of great value to further explore the effect of scarcity on panic buying during public emergencies, few studies have concentrated on this perspective. Our study seems to bridge this gap for the first time.

It is worth mentioning that during our preparation of this article, the work of Islam et al. (2020) was published. They also focused on the aggravating effect of scarcity on “panic buying” through psychological arousal during the COVID-19 outbreak. However, their study used impulsive and obsessive buying as indicators of “panic buying,” both of which are essentially different from panic buying (Yuen et al., 2020) and excessive acquisition (Cannito et al., 2021). Based on the academic definition of panic buying and its performance in real life, our study evaluated panic buying from two aspects, namely, the increase of consumption quantity and the increase of consumption price, thus employing hoarding and payment degree as the indicators. This approach better reflects the essence of panic buying and improves the stability of the results. Moreover, our study employed various manifestations of scarcity, which enhanced the robustness of the results (Piff et al., 2010). The results implied that not only subjective scarcity but also perceived scarcity should be considered during public emergencies. When objective scarcity is difficult to alleviate, we can start with subjective scarcity in order to reduce panic buying or other undesirable mindsets.

Second, drawing on the standard learning hierarchical model of consuming decisions (Lee and Goudeau, 2014) and the cognition-affect-coping model of coping behaviors (Jung and Park, 2018), we determined that the link between scarcity and panic buying is transmitted *via* reduced levels of perceived control and enhanced levels of panic, especially in life-threatening crises such as public health emergencies.

As vital psychological variables that impact individual behaviors during public emergencies, perceived control and panic have been found to have independent impacts on panic buying (Sterman and Dogan, 2015; Garbe et al., 2020; Yuen et al., 2020; Bentall et al., 2021); however, few studies have investigated their roles in the relationship between scarcity and panic buying. Our results suggested that perceived control and panic serially mediate the effect of scarcity on panic buying. This serial mediation is a deeper and more stable mechanism of the link between scarcity and panic buying than the independent mediation effects of panic and perceived control. At the same time, the “perceived control → panic” pathway was also previously found to affect individual emotion regulation consumption in disasters (Kemp et al., 2014). These findings implicated the understanding of individuals’ consumption behavior during public emergencies from a more comprehensive perspective integrating cognition and affect. Furthermore, the serial mediating pathway suggested, in practice of emergency management, effective policies which compensate perceived control and relive panic subsequently, such as price control, are essential to lessen panic buying.

On the other hand, the serial mediating pathway has boundaries. The pathway is more suitable for public health emergencies such as epidemics and pollutant leakage compared to situations with lower levels of risk. In a public emergency with lower risk, higher familiarity and higher predictability, such as hurricanes, perceived control and panic cannot account for the aggravating effect of scarcity on panic buying. A previous study found a “perceived control → fear/anxiety → hedonic rationalizations → emotion regulation consumption” pattern in hurricanes (Kemp et al., 2014). Integrating our results with those of the previous work reminds us that during public emergencies such as hurricanes, scarcity may impact panic buying through other processes, while perceived control and panic may influence other consumption behaviors.

There are still some limitations of this research. First, the capital (i.e., financial, social, cultural) or other production inputs (i.e., time) that the consumer invests in order to acquire and use goods and services is another type of scarcity found in consumer decision journeys (Hamilton et al., 2019). Whereas the current study mainly focused on product scarcity, future studies can continue to explore the effect of capital scarcity on panic buying and its interaction with product scarcity on panic buying. Second, we used information containing relevant keywords as the objective indicator of scarcity and panic buying in Study 1. Although we did our best to reduce the noise of the information to ensure the highest relevance, more objective and more direct indicators such as supply data and consumer data from authority agencies is needed for future studies. Finally, more investigations on the understanding of the “black box” of the effect of scarcity on panic buying are needed. On the one hand, future research can continue to explore the combination of other psychological variables under the current “cognition-affect” framework to expand the psychological mechanism of the link between scarcity and panic buying. On the other hand, while the current research has mainly focused on the generalization of the serial mediation model in various types of public emergencies, some individual characteristics, as they could affect buying pattern directly or indirectly, should be considered in the future. For example, studies indicated some personality traits (e.g., Yoshino et al., 2021) and the psychological need for necessities products (Di Crosta et al., 2021) were positively correlated to excessive acquisition and spending level. Moreover, health anxiety (HA) predicted attentional bias toward virus-related stimuli (Cannito et al., 2020), and hoarding level affected temporal discounting of mask (Cannito et al., 2021), suggesting HA and hoarding level could influence the relation between scarcity and panic buying they could change individuals’ buying pattern. At the same time, the current research has not verified the roles of perceived control and panic in the effect of scarcity on panic buying in emergencies with lower risk, such as hurricanes. This lack of verification suggests that more studies are needed to clarify the effect of scarcity in panic buying in such a context.

Taken together, the present study is an initial attempt to explore the cause of panic buying during public emergencies from the perspective of scarcity. Moreover, it puts forward the cognition-affect serial pathway to interpret the link between scarcity and panic buying during public emergencies, which integrates a comprehensive formwork of the contextual and psychological antecedents of external behaviors and examines the boundaries of this pathway. This study enriches the relevant research on panic buying and provides some practical guidance for the social management of public emergencies, especially public health emergencies.

## Data Availability Statement

The raw data supporting the conclusions of this article will be made available by the authors, without undue reservation.

## Ethics Statement

The studies involving human participants were reviewed and approved by Institutional Review Board for Human Participants at Tsinghua University. The patients/participants provided their online informed consent to participate in this study.

## Author Contributions

JL developed the idea, organized the studies, and revised the manuscript. XM collected and analyzed the data, and drafted the manuscript. Both authors contributed to the article and approved the submitted version.

## Conflict of Interest

The authors declare that the research was conducted in the absence of any commercial or financial relationships that could be construed as a potential conflict of interest.

## Publisher’s Note

All claims expressed in this article are solely those of the authors and do not necessarily represent those of their affiliated organizations, or those of the publisher, the editors and the reviewers. Any product that may be evaluated in this article, or claim that may be made by its manufacturer, is not guaranteed or endorsed by the publisher.

## Footnotes

1 QuestMobile. (2020-02-12). *2020 China mobile Internet “Fighting epidemic” Special Report – Hot industry development Report (in Chinese)*. https://www.questmobile.com.cn/research/report-new/81

2 WeChat is one of the most popular multi-purpose messaging APPs in mainland China and users can express their views through WeChat’s official accounts which are similar to blogs. Weibo is one of the most popular social media in China, which is similar to Facebook and Twitter. TouTiao and Sohu are the two most active news and information platforms in Chinese mainland. Other online platforms refers to some small online information platforms and the platforms which cannot be categorized into the former groups.

3
yuqing.gsdata.cn

4 Ministry of Industry and Information Technology of the People’s Republic of China. (2020-02-14). List of key supplies for epidemic prevention and control (medical emergency). https://www.miit.gov.cn/ztzl/rdzt/xxgzbdgrdfyyqfkgz/tzgg/art/2020/art_8ac3f70d70a14e8abbce47d08bdbfd8d.html

5
https://www.wjx.cn

6 The distance from the leakage of radioactive pollutants in real life was measured by familiarity with this kind of events (1 = very unfamiliar, 7 = very familiar).

7 The Cronbach’s α of items assessing panic was 0.88.

8 Significant differences among the risk assessment of the emergencies in studies 3a–3c were found using items developed by Xie et al. (2005) [*F*(2,1145) = 34.88, *p* < 0.001, η_*p*_^2^ = 0.06]. Specifically, participants perceived lower risk in the hurricane situation (*M* = 4.49, *SD* = 1.34) than that in epidemic situation (*M* = 5.08, *SD* = 1.29) and leakage situation (*M* = 5.17, *SD* = 1.31) (*ps* < 0.001), whereas there was no significant difference between the latter two (*p* = 0.312). Moreover, there were significant differences in panic among the three emergencies, *F*(2,1145) = 21.54, *p* < 0.001, η_*p*_^2^ = 0.04. Panic in hurricane situation (*M* = 2.84, *SD* = 0.83)was lower than that in epidemic (*M* = 3.02, *SD* = 0.86) and leakage (*M* = 3.22, *SD* = 0.82) (*ps* < 0.001).
