# Supplementary material for: Buying to Cope With Scarcity During Public Emergencies: A Serial Mediation Model Based on Cognition-Affect Theory
Source: Front Psychol. 2022 Jan 27;12:791850. doi: 10.3389/fpsyg.2021.791850 (PMC8828481; doi:10.3389/fpsyg.2021.791850)
Supplement: Supplementary file 1 [file Table_1.DOCX]

Supplementary materials

1. Measuring materials in Study 2

(1) Perceived Scarcity

Scarcity refers to what we have is less than what we need. Please indicate to what extent you feel scarce on the resource related to the present pandemic. 1 = very sufficient, 2 = sufficient, 3 = sufficient somewhat, 4 = neutral, 5 = scarce somewhat, 6 = scarce, 7 = very scarce

| Item | 1 | 2 | 3 | 4 | 5 | 6 | 7 |
| --- | --- | --- | --- | --- | --- | --- | --- |
| A. The prevention measures against COVID-19 I adopt |  |  |  |  |  |  |  |
| B. The prevention equipment against COVID-19 I have |  |  |  |  |  |  |  |
| C. The knowledge about epidemic prevention I know |  |  |  |  |  |  |  |
| D. The useful information about COVID-19 I know |  |  |  |  |  |  |  |

(2) Perceived Control

Please indicate to what extent you agree with each statement below. 1 = strongly disagree, 2 = disagree somewhat, 3 = agree somewhat, 4 = strongly agree.

| Item | 1 | 2 | 3 | 4 |
| --- | --- | --- | --- | --- |
| A. There isn’t much I can do to help myself feel better about the present pandemic. |  |  |  |  |
| B. How I deal with the present pandemic now is under my control. |  |  |  |  |
| C. I don’t have much control over my emotional reactions to the present pandemic. |  |  |  |  |
| D. When I am upset about the present pandemic, I can find a way to feel better. |  |  |  |  |
| E. I have control over my day-to-day reactions to the present pandemic. |  |  |  |  |
| F. There isn’t much I can do to keep the present pandemic from affecting me.  G. I have control over how I think about the present pandemic.  H. My reaction to the present pandemic is not under my control. |  |  |  |  |

(3) Panic

Please indicate to what extent you have felt each of the following emotions since the outbreak of the current pandemic. 1= very slight or not at all, 2 = a little, 3 = moderately, 4 = quite a bit, 5 = extremely.

| Item | 1 | 2 | 3 | 4 | 5 |
| --- | --- | --- | --- | --- | --- |
| A. scared |  |  |  |  |  |
| B. afraid |  |  |  |  |  |
| C. nervous |  |  |  |  |  |
| D. jittery |  |  |  |  |  |

(4) Panic buying

You will see some pictures of the epidemic prevention product. Please indicate the highest price you are willing to pay for the product at present.

A.
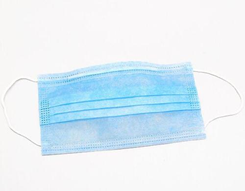
 _____ RMB yuan per piece

B.
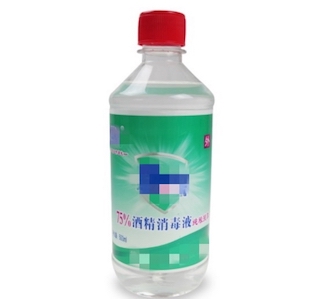
 _____ RMB yuan per bottle (500 ml)

C.
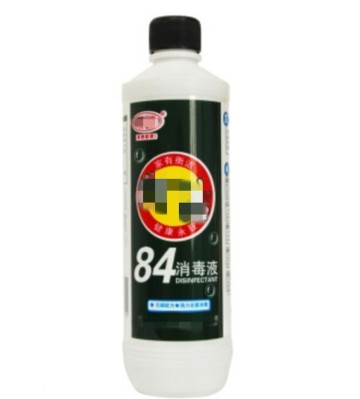
 _____ RMB yuan per bottle (500 ml)

D.
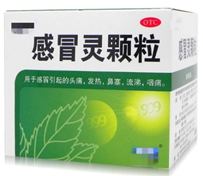
 _____ RMB yuan per box (500 ml)

2. Exploratory factor analysis (EFA) of the measure of perceived scarcity in Study 2

We examined the factor structure of the measure of perceived scarcity in Study 2 using EFA through SPSS 26.0. Before conducting the EFA of the 4 items, we assessed the factorability of the items. The Kaiser-Meyer-Olkin (KMO) test statistic (.73) and the Bartlett’s test of sphericity (Approximate χ^2^= 642.41, df = 6, p < 0.001) indicated the items were appropriate for factor analysis (Dziuban & Shirkey, 1974; Tobias & Carlson, 1969). In conducting this EFA, we used principal component analysis as the extraction method and a varimax rotation. The result showed that all items loaded clearly on one factor, and this one-factor structure explained 57.57% of the total variance. Table S1 showed specific factor loading of each item.

Table S1. Pattern matrix of the PCA for the measurements of perceived scarcity in Study 2

| Item | Factor loadings |
| --- | --- |
| 1. The prevention measures against COVID-19 I adopt | .76 |
| 2. The prevention equipment against COVID-19 I have | .68 |
| 3. The knowledge about epidemic prevention I know | .83 |
| 4. The useful information about COVID-19 I know | .76 |

References:

Dziuban, C. D., & Shirkey, E. C. (1974). When is a correlation matrix appropriate for factor analysis? *Psychological Bulletin, 81*, 358-361.

Tobias, S., & Carlson, J. E. (1969). Brief report: Bartlett's test of sphericity and chance findings in factor analysis. Multivariate Behavioral Research, 4(3), 375-377.

3. Experiment materials in Study 3

3.1 Priming materials

(1) Study 3a

Suppose that a new respiratory infectious disease has broken out in the country where you live. This disease spreads through droplets, air and direct contact. At present, many people have been infected, and the number of infected people is still rising. Because there are asymptomatic infected people, it is impossible to determine the scale of potential infected people in the population, and the mortality rate of infected people is about 10%. Specific drugs and vaccines are still under development. At present, people can only protect themselves through some protective products, such as surgical masks, medical alcohol and some medicine with certain preventive functions, and prepare some basic food for daily life.

(Scarcity group) Because the virus is a new virus, there is little information about it, and there are few channels for you to know about it. Due to the sharp increase in the demand, the protective products in the market are being out of stock, and the supply is difficult in the near future.

(Non-Scarcity group) Because the virus is a variation of a known virus, there is a lot of information about it, and people have a lot of channels to get the information. Besides, the marketing stock of the protective products is sufficient now, and the supply and demand are stable. Factories are in rush production to meet the public demand.

(2) Study 3b

Suppose there is a sudden leakage of pollutants in the city where you are located. The pollutants contain carcinogens such as DDT and polychlorinated biphenyls. Soil, surface rivers and groundwater are polluted. At present, pollution is still spreading, and the final pollution scope cannot be determined.

(Scarcity group) Because the incident happened suddenly, there is little information about it, and there are few channels for you to know the relevant information. As the leakage of pollutants directly affects people's lives, the demand for necessities of life such as bottled drinking water, fruits and vegetables, basic food, and some medicine with the detoxification function has increased sharply. And some people also choose to go to other cities to avoid the impact of the accident. At present, the necessities products and tickets are in face of being out of stock, and the subsequent supply is difficult.

(Non-Scarcity group) At present, there is a lot of information about the incident, and there are many channels for you to know the relevant information. As the leakage of pollutants directly affects people's lives, some necessities of life such as bottled drinking water, fruits and vegetables, basic food, and some medicine with detoxification function are needed. And some people also choose to go to other cities to avoid the impact of the accident. The supply of these necessities products is as usual, and the stock of them is still sufficient now.

(3) Study 3c

Suppose that the city where you live is a small seaside town. According to the weather prediction, the strong hurricane "Florence" is moving rapidly and is expected to land in the city in three days and reach level 4 (up to level 5). The hurricane will bring heavy rainfall and cause serious floods, damage roads, power systems, water supply systems and other infrastructure, and greatly affect people's lives.

(Scarcity group) The relevant information released by the meteorological department is limited now, and there are few channels for you to know the relevant information. As the hurricane will directly affect people's lives, the demand for the necessities for life such as bottled drinking water, fruits and vegetables, basic food and flashlights has increased sharply. And some people also choose to go to other cities to avoid the impact of the hurricane. At present, the necessities products and tickets are in face of being out of stock and the subsequent supply is difficult.

(Non-Scarcity group) The meteorological department releases a lot of relevant information now, and there are many channels for you to know relevant information. As hurricanes will directly affect people's lives, the necessities for life such as bottled drinking water, fruits and vegetables, basic food and flashlights are needed. And some people also choose to go to other cities to avoid the impact of the hurricane. The supply of these necessities products is as usual, and the stock of them is still sufficient now.

3.2 Panic buying

(1) Study 3a

A. Hoarding

In the above situation, how many bottles of medical alcohol (300 ml/bottle) will you buy? (Please fill in an integer)

In the above situation, how many pieces of surgical mask will you buy? (Please fill in an integer)

In the above situation, how many boxes of medicine with certain preventive functions will you buy? (Please fill in an integer)

In the above situation, how many days of food will you prepare? (Please fill in an integer)

B. Payment degree

The daily unit price of the medical alcohol is 9 yuan. In the above situation, what is the highest unit price you are willing to pay?

The daily unit price of the surgical mask is 10 yuan / piece. In the above situation, what is the highest unit price you are willing to pay?

The daily unit price of the medicine with certain preventive functions is 14 yuan. In the above situation, what is the highest unit price you are willing to pay?

How much are you willing to pay more than usual on food in the above situation?

(2) Study 3b & Study 3c

A. Hoarding

How many days of bottled drinking water do you plan to buy more than usual?

How many days of fruits and vegetables do you plan to buy more than usual?

How many days of food do you plan to buy more than usual?

How many boxes of the medicine with the detoxification function do you plan to buy more than usual? (Study 3b)

B. Payment degree

How much more are you willing to pay than usual on bottled drinking water in the above situation? (Please fill in a percentage)

How much more are you willing to pay than usual on fruits and vegetables in the above situation? (Please fill in a percentage)

How much more are you willing to pay than usual on food in the above situation? (Please fill in a percentage)

How much more are you willing to pay than usual on the medicine with the detoxification function in the above situation? (Please fill in a percentage) (Study 3b)

How much more are you willing to pay than usual on a flashlight in the above situation? (Please fill in a percentage) (Study 3c)

How much more are you willing to pay than usual on a ticket to other cities in the above situation? (Please fill in a percentage)

3.3 Manipulation check

How would you describe the quantity of the protective supplies in the above situation?

1 (very sufficient) -------------7 (very insufficient)

How would you describe the quantity of the information related to epidemic in the above situation?

1 (very sufficient) -------------7 (very insufficient)

4. Supplementary analysis of

**Table S2.** Summary of indirect effects in study 3a

| Indirect effects | Effect (SE) | 95%CI |
| --- | --- | --- |
| *Payment degree* |  |  |
| Total (for serial mediation model) | 0.08 (0.06) | [-0.02, 0.20] |
| scarcity → perceived control → panic buying | 0.03 (0.03) | [-0.015, 0.12] |
| scarcity → panic → panic buying | 0.04 (0.03) | [-0.01, 0.11] |
| scarcity → panic → perceived control → panic buying | 0.01(0.01) | [-0.003, 0.04] |
| scarcity → perceived control → panic buying (single mediation model) | 0.05 (0.04) | [-0.03, 0.14] |
| scarcity → panic → panic buying (single mediation model) | 0.07 (0.04) | [-0.002, 0.16] |
| scarcity → perceived control → panic (single mediation model) | 0.15 (0.06) | [0.05, 0.28] |
| perceived control → panic → panic buying (single mediation model) | -0.05 (0.02) | [-0.10, -0.02] |
| *Hoarding* |  |  |
| Total (for serial mediation model ) | 0.20 (0.07) | [0.08, 0.35] |
| scarcity → perceived control → panic buying | 0.004 (0.04) | [-0.07, 0.10] |
| scarcity → panic → panic buying | 0.16 (0.05) | [0.07, 0.27] |
| scarcity → panic → perceived control → panic buying | 0.04 (0.02) | [0.01, 0.09] |
| scarcity → perceived control → panic buying (single mediation model) | 0.05 (0.05) | [-0.04, 0.15] |
| scarcity → panic → panic buying (single mediation model) | 0.20 (0.06) | [0.11, 0.32] |
| scarcity → perceived control → panic (single mediation model) | 0.15 (0.06) | [0.05, 0.28] |
| perceived control → panic → panic buying (single mediation model) | -0.11 (0.03) | [-0.17, -0.05] |

**Table S3.** Summary of indirect effects in study 3b

| Indirect effects | Effect (SE) | 95%CI |
| --- | --- | --- |
| *Payment degree* |  |  |
| Total (for serial mediation model ) | 0.20 (0.07) | [0.08, 0.34] |
| scarcity → perceived control → panic buying | 0.06 (0.06) | [-0.05, 0.18] |
| scarcity → panic → panic buying | 0.08 (0.03) | [0.02, 0.15] |
| scarcity → panic → perceived control → panic buying | 0.07 (0.02) | [0.03, 0.13] |
| scarcity → perceived control → panic buying (single mediation model) | 0.13 (0.06) | [0.02, 0.25] |
| scarcity → panic → panic buying (single mediation model) | 0.17 (0.05) | [0.08, 0.27] |
| scarcity → perceived control → panic (single mediation model) | 0.33 (0.07) | [0.20, 0.47] |
| perceived control → panic → panic buying (single mediation model) | -0.12 (0.02) | [-0.16, -0.07] |
| *Hoarding* |  |  |
| Total (for serial mediation model ) | 0.14 (0.06) | [0.02, 0.27] |
| scarcity → perceived control → panic buying | 0.01(0.06) | [-0.10, 0.11] |
| scarcity → panic → panic buying | 0.07 (0.03) | [0.02, 0.14] |
| scarcity → panic → perceived control → panic buying | 0.06 (0.02) | [0.02, 0.12] |
| scarcity → perceived control → panic buying (single mediation model) | 0.07 (0.06) | [-0.04, 0.19] |
| scarcity → panic → panic buying (single mediation model) | 0.14 (0.05) | [0.05, 0.23] |
| scarcity → perceived control → panic (single mediation model) | 0.33 (0.07) | [0.20, 0.47] |
| perceived control → panic → panic buying (single mediation model) | -0.10 (0.03) | [-0.16, -0.05] |

**Table S4.** Summary of indirect effects in study 3c

| Indirect effects | Effect (SE) | 95%CI |
| --- | --- | --- |
| *Payment degree* |  |  |
| Total (for serial mediation model) | -0.01 (0.04) | [-0.09, 0.06] |
| scarcity → perceived control → panic buying | -0.03 (0.04) | [-0.11, 0.04] |
| scarcity → panic → panic buying | 0.004 (0.01) | [-0.01, 0.03] |
| scarcity → panic → perceived control → panic buying | 0.01 (0.01) | [-0.02, 0.04] |
| scarcity → perceived control → panic buying (single mediation model) | -0.02 (0.04) | [-0.10, 0.05] |
| scarcity → panic → panic buying (single mediation model) | 0.01 (0.02) | [-0.04, 0.05] |
| scarcity → perceived control → panic (single mediation model) | 0.25 (0.06) | [0.13, 0.38] |
| perceived control → panic → panic buying (single mediation model) | -0.03 (0.03) | [-0.08, 0.02] |
| *Hoarding* |  |  |
| Total (for serial mediation model ) | -0.07 (0.04) | [-0.16, 0.02] |
| scarcity → perceived control → panic buying | -0.05 (0.05) | [-0.14, 0.04] |
| scarcity → panic → panic buying | -0.01 (0.01) | [-0.04, 0.02] |
| scarcity → panic → perceived control → panic buying | -0.01 (0.02) | [-0.06, 0.02] |
| scarcity → perceived control → panic buying (single mediation model) | -0.06 (0.04) | [-0.15, 0.02] |
| scarcity → panic → panic buying (single mediation model) | -0.03 (0.03) | [-0.09, 0.02] |
| scarcity → perceived control → panic (single mediation model) | 0.25 (0.06) | [0.13, 0.38] |
| perceived control → panic → panic buying (single mediation model) | 0.02 (0.03) | [-0.05, 0.08] |
